# Functional Dissection of the Blocking and Bypass Activities of the *Fab-8* Boundary in the *Drosophila* Bithorax Complex

**DOI:** 10.1371/journal.pgen.1006188

**Published:** 2016-07-18

**Authors:** Olga Kyrchanova, Vladic Mogila, Daniel Wolle, Girish Deshpande, Alexander Parshikov, Fabienne Cléard, Francois Karch, Paul Schedl, Pavel Georgiev

**Affiliations:** 1 Department of Genetics, Institute of Gene Biology, Russian Academy of Sciences, Moscow, Russia; 2 Department of Molecular Biology, Princeton University, Princeton, New Jersey, United States of America; 3 Department of Genetics and Evolution, University of Geneva, Geneva, Switzerland; Centro de Biología Molecular Severo Ochoa (C.S.I.C.-U.A.M.), SPAIN

## Abstract

Functionally autonomous regulatory domains direct the parasegment-specific expression of the *Drosophila* Bithorax complex (BX-C) homeotic genes. Autonomy is conferred by boundary/insulator elements that separate each regulatory domain from its neighbors. For six of the nine parasegment (PS) regulatory domains in the complex, at least one boundary is located between the domain and its target homeotic gene. Consequently, BX-C boundaries must not only block adventitious interactions between neighboring regulatory domains, but also be permissive (bypass) for regulatory interactions between the domains and their gene targets. To elucidate how the BX-C boundaries combine these two contradictory activities, we have used a boundary replacement strategy. We show that a 337 bp fragment spanning the *Fab-8* boundary nuclease hypersensitive site and lacking all but 83 bp of the 625 bp *Fab-8* PTS (promoter targeting sequence) fully rescues a *Fab-7* deletion. It blocks crosstalk between the *iab-6* and *iab-7* regulatory domains, and has bypass activity that enables the two downstream domains, *iab-5* and *iab-6*, to regulate *Abdominal-B* (*Abd-B*) transcription in spite of two intervening boundary elements. *Fab-8* has two dCTCF sites and we show that they are necessary both for blocking and bypass activity. However, CTCF sites on their own are not sufficient for bypass. While multimerized dCTCF (or Su(Hw)) sites have blocking activity, they fail to support bypass. Moreover, this bypass defect is not rescued by the full length PTS. Finally, we show that orientation is critical for the proper functioning the *Fab-8* replacement. Though the inverted *Fab-8* boundary still blocks crosstalk, it disrupts the topology of the *Abd-B* regulatory domains and does not support bypass. Importantly, altering the orientation of the *Fab-8* dCTCF sites is not sufficient to disrupt bypass, indicating that orientation dependence is conferred by other factors.

## Author Summary

Boundary elements in the Bithorax complex have two seemingly contradictory activities. They must block crosstalk between neighboring regulatory domains, but at the same time be permissive (insulator bypass) for regulatory interactions between the domains and the BX-C homeotic genes. We have used a replacement strategy to investigate how they carry out these two functions. We show that a 337 bp fragment spanning the *Fab-8* boundary nuclease hypersensitive site is sufficient to fully rescue a *Fab-7* boundary deletion. It blocks crosstalk and supports bypass. As has been observed in transgene assays, blocking activity requires the *Fab-8* dCTCF sites, while full bypass activity requires the dCTCF sites plus a small part of PTS. In transgene assays, bypass activity typically depends on the orientation of the two insulators relative to each other. A similar orientation dependence is observed for the *Fab-8* replacement in BX-C. When the orientation of the *Fab-8* boundary is reversed, bypass activity is lost, while blocking is unaffected. Interestingly, unlike what has been observed in mammals, reversing the orientation of only the *Fab-8* dCTCF sites does not affect boundary function. This finding indicates that other *Fab-8* factors must play a critical role in determining orientation. Taken together, our findings argue that carrying out the paradoxical functions of the BX-C boundaries does not require any unusual or special properties; rather BX-C boundaries utilize generic blocking and insulator bypass activities that are appropriately adapted to their regulatory context. Thus making them a good model for studying the functional properties of boundaries/insulators in their native setting.

## Introduction

Special elements called chromatin boundaries or insulators are thought to subdivide chromosomes in multi-cellular eukaryotes into topologically and genetically autonomous domains [1–10]. Boundaries/insulators have both architectural and genetic functions. The architectural functions depend upon physical interactions between insulators. The first indication that boundary elements interact with each other came from the discovery that insulators can facilitate regulatory interactions between transgenes inserted at distant sites [11–16]. Subsequent work confirmed that the long distance regulatory interactions involved direct physical contacts between boundaries [17,18]. Moreover, it was shown that these physical interactions provide the anchors for the formation of topologically independent loops [7,9,19–21].

In addition to subdividing the chromosome into a series of looped domains, insulators have a number of genetic functions. These functions have been most thoroughly documented using transgene assays and include enhancer/silencer blocking and bypass activities [6,22]. In blocking assays, boundaries prevent regulatory interactions when interposed between enhancers or silencers and a reporter gene [23–25]. This insulation activity is position dependent, and boundaries do not block when the enhancers/silencers are located in between the reporter and the boundary. In bypass assays, two boundaries (instead of one) are interposed between an enhancer or silencer and a reporter gene [26–28]. When the two boundaries are appropriately matched and correctly oriented, they pair with each other in a manner that brings the enhancers/silencers into contact with the reporter [29,30].

**Fig 1:**
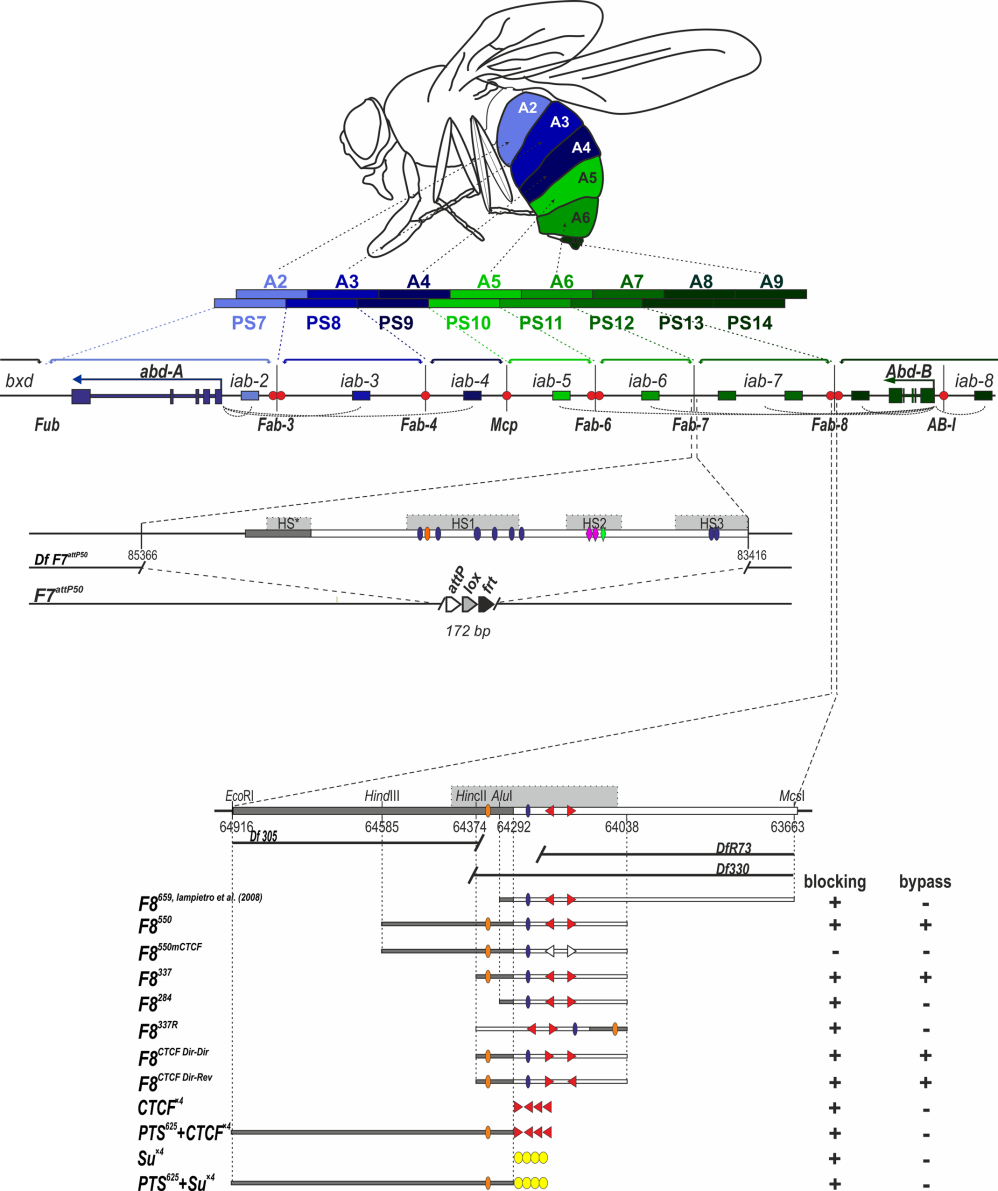
Fragments of *Fab-8* used for *Fab-7* replacement. (A) Regulatory region of the distal part of the BX-C. Horizontal arrows represent transcripts for *abd-A* (blue) and *Abd-B* (green). *iab* enhancers are shown as rectangles color-coded to the respective gene they control (darker shades of color indicate higher expression levels). The dotted arches are a graphical illustration of the targeting of each *cis*-regulatory domain to the *abd-A* or *Abd-Bm* promoter. Vertical lines mark boundaries (*Fub*, *Fab-3*, *Fab-4*, *Mcp*, *Fab-6*, *Fab-7*, and *Fab-8*) of regulatory *iab* domains which are delimited by brackets above the map. There is also a boundary-like element *AB-I* upstream of the *Abd-B* promoter that has communicator activity in bypass assays. CTCF binding sites at boundaries are shown as red circles. Abdominal segments (A2-9) and parasegments (PS7-14) that correspond to the *iab* domains of *abd-A* and *Abd-B* are shown as multicolored bars. Localization of the segments in adult male is shown on the fly drawing at the top. Deletion of *Fab-7* in *Fab-7*^*attP50*^ is shown separately. (B) Molecular maps of the *Fab-8* boundary replacement constructs analyzed in the paper and their blocking and bypass activities. *Fab-8* insulator is shown as a horizontal bar. PTS is marked by dark gray. DNase hypersensitive site is shown as a light gray box above the coordinate bar. The proximal and distal deficiency endpoints of the *Fab-8* deletions are shown below. Known protein binding sites are indicated. Binding factors, common for *Fab-7* and *Fab-8*, are shown as ovals: blue–GAF, orange–Elba/Insv. The non-common factors for *Fab-7* –as rhombi: rose–Pita, green–Zipic. dCTCF binding sites are shown as red triangles indicating orientation of the sites. Empty triangles mark the mutated sites. Su(Hw) binding sites are denoted as yellow ovals. On the right side of the constructs, the blocking and bypass activities of each replacement construct are shown.

While all of the fly boundaries that have been tested have blocking and bypass activities in transgene assays, it is not clear to what extent these activities are important or relevant in their endogenous settings, or how they are related to each other. For example, boundary elements are known to play a central role in the parasegment-specific regulation of the three BX-C Hox genes, *Ultrabithorax* (*Ubx*), *abdominal-A* (*abd-A*), and *Abdominal-B* (*Abd-B*) [31,32]. However, the functions of the BX-C boundaries in the context of the complex appear, at least on the surface, to be rather different from those detected in transgene assays. The differences are most clearly elaborated for the boundaries associated with the four regulatory domains, *iab-5*, *iab-6*, *iab-7*, and *iab-8*, that control *Abd-B* expression in parasegments PS10, PS11, PS12, and PS13, respectively (Fig 1A). In order to specify PS identity, each of these regulatory domains must be able to function autonomously. Genetic and molecular studies have shown that boundary elements (*Mcp*, *Fab-6*, *Fab-7*, and *Fab-8*; see Fig 1A) bracket each regulatory domain, and that one of their key functions is to ensure autonomous activity [33–44]. The most thoroughly characterized BX-C boundary, *Fab-7*, is located between *iab-6* and *iab-7* (Fig 1A). *Fab-7* deletions fuse the *iab-6* and *iab-7* regulatory domains and they exhibit a complex mixture of gain- (GOF) and loss-of-function (LOF) phenotypes in PS11 [38,40]. The GOF phenotypes arise because *iab-6* initiator inappropriately activates *iab-7* in PS11, while the LOF phenotypes arise because repressive elements in *iab-7* that are active in PS11 silence *iab-6* in that parasegment [45,46]. A similar fusion of neighboring regulatory domains and a consequent misregulation of *Abd-B* is observed when *Fab-6* and *Fab-8* are deleted [34,42]. Though these BX-C boundaries can also block enhancers and silencers from regulating a reporter gene in transgene assays, this type of blocking activity is not directly relevant to the normal biological functions of these elements in the complex [34,47–55]. In BX-C, boundaries ensure autonomy by preventing crosstalk between initiation elements, enhancers, and silencers in the adjacent domains, not by blocking these elements from regulating the activity of promoters. As this is a role that may be unique to BX-C, it would be reasonable to think that the mechanisms and factors used to block crosstalk between regulatory elements in adjacent domains might be rather different from those that are needed to prevent enhancers or silencers from influencing RNA Pol II transcription.

Several observations have reinforced the idea that BX-C boundaries have properties that distinguish them from boundaries elsewhere in the fly genome and in other eukaryotes. Six of the regulatory domains in BX-C (including three for *Abd-B*) are separated from their target genes by at least one boundary element. Since the tissue-specific regulatory elements in these domains are still able to regulate their respective target genes, the BX-C boundaries must be permissive for interactions between the domains and the transcriptional machinery at the promoters of the three BX-C Hox genes. A plausible mechanism for bypassing BX-C boundaries came from the discovery that *Fab-7* and *Fab-8* have special promoter targeting sequences that can facilitate enhancer-promoter interactions in transgene assays [56,57]. While non-BX-C boundaries can also bring distant enhancers and promoters together in the insulator bypass assay, this activity requires two appropriately matched boundaries and is non-autonomous. By contrast, the PTS elements associated with *Fab-7* and *Fab-8* appeared to function autonomously in transgene assays. Further evidence that BX-C boundaries are distinct from generic insulators was provided by *Fab-7* replacement experiments using *su(Hw)* and *scs* [58]. While both blocked crosstalk between *iab-6* and *iab-7*, these two insulators clearly differed from BX-C boundaries in that they also prevented the downstream *iab-6* regulatory domain from regulating *Abd-B*.

In the studies reported here, we have asked what boundary functions are actually needed in the context of BX-C. For this purpose, we have replaced *Fab-7* with the neighboring boundary, *Fab-8*. The *Fab-8* replacement we used includes part of the PTS and it fully rescues a *Fab-7*^*attP50*^ deletion. Our subsequent functional dissection indicates that the *Fab-8* boundary is able to substitute for Fab-7 because its entirely generic boundary activities (blocking and bypass) are appropriately matched to its neighborhood.

## Results

### A *Fab-8* fragment spanning the nuclease hypersensitive region fully substitutes for *Fab-7*

In previous studies, Iampietro et al. [59] attempted to rescue a *Fab-7* deletion with a 659 bp fragment containing *Fab-8* sequences (Fig 1B). While they found that this *Fab-8* fragment blocked crosstalk between *iab-6* and *iab-7*, it was unable to fully support bypass. The 659 bp fragment used by Iampietro et al. lacked a ~100 bp sequence from the centromere proximal side of the *Fab-8* nuclease hypersensitive region. There were several reasons to think that this sequence from the hypersensitive region, or even more centromere proximal sequences might be important for *Fab-8* function. One came from the characterization of the *Fab-8* deletion mutant, *iab-7*^*R73*^, which removes sequences from the centromere proximal side of the *Fab-8* boundary [41]. *iab-7*^*R73*^ has a weak LOF phenotype in PS12. One explanation for this phenotype is that the deleted sequences are required for bypass activity. Two findings are consistent with this possibility. First, *iab-7*^*R73*^ removes the PTS that in transgene assays can direct enhancer sequences to a promoter [56]. Second, in bypass assays these same PTS sequences are necessary, but not in themselves sufficient to support interactions between *Fab-8* and itself, and between *Fab-8* and either *Fab-7* or an insulator-like element, *AB-I*, located upstream of the *Abd-B* promoter [30,60]. Finally, the *iab-7*^*R73*^ deletion extends into the proximal half of the nuclease hypersensitive region of *Fab-8*, and the region of overlap contains binding sites for two factors known to be involved in the insulator activity of the adjacent *Fab-7* boundary, Elba and LBC [34,61,62].

**Fig 2:**
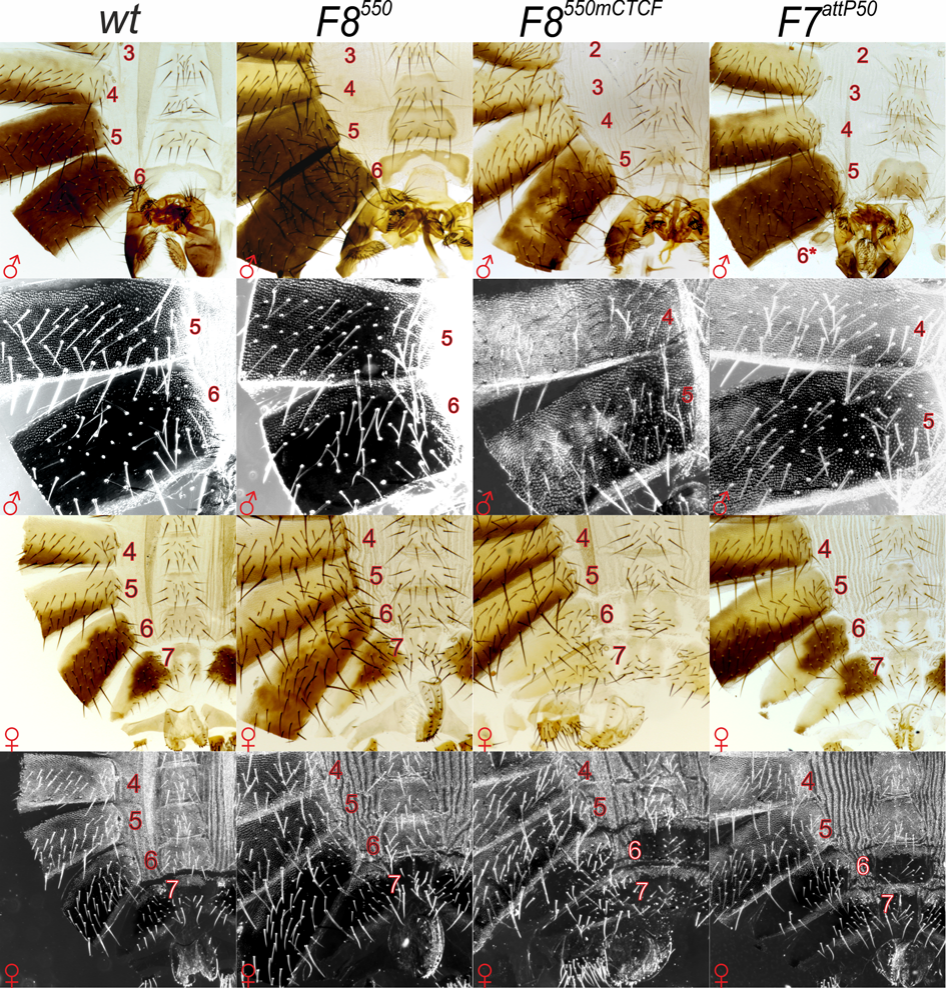
*Fab-8*^*550*^ is able to substitute for *Fab-7*. Morphology of the 5th to 8th abdominal segments (numbered) is determined by the *Abd-B cis*-regulatory regions. Wild-type (*wt*). Males: the 5th and 6th tergites are pigmented, the A6 sternite is recognizable by the absence of bristles and a specific form; A7 does not contribute to any visible cuticle structures. Trichomes are visible in the dark field and cover all the surface of A5 tergite and only a thin stripe along the anterior and ventral edges of the A6 tergite. Females: the 6th tergite is almost completely pigmented, dorsal part of the A7 tergite is depigmented, A7 sternite (ventral) has a characteristic shape with large bristles pointing towards the posterior; A8 tergite is the smallest one with no pigmentation, bristles, or trichomes. In dark field: the entire surface of the A5 tergite is evenly covered by trichomes, most of the A6 and A7 tergites is devoid of trichomes, except the anterior edges, and the ventral edge in A7. *F8*^*550*^ resembles wild type. *F8*^*550mCTCF*^ have mixed gain- and loss-of-function (GOF-LOF) phenotype. In males, A6 disappears completely (strong GOF transformation) but at the same time A5 acquires some features of A4 (mosaic LOF phenotype indicating a defect in the functioning of *iab-5*). Females have GOF phenotypes in A6 and A7 that are not observed in either *Fab-7* boundary deletions or in deletions that remove both the *Fab-7* boundary and the HS3 *iab-7* PRE (compare *F8*^*550mCTCF*^ females with *F7*^*attP50*^). These include a reduction in size, an almost complete loss of pigmentation of the tergite and an abnormal pattern of bristles in the sternite. *F7*^*attP50*^ males and females have the classic GOF transformation of A6 (PS11) into A7 (PS12) seen in mutations that remove both the *Fab-7* boundary and the HS3 *iab-7* PRE.

Since studies on other insulators indicate that critical sequences often map to hypersensitive regions, we re-centered the *Fab-8* fragment, *F8*^*550*^, used for replacement, so that it spanned the entire nuclease hypersensitive region, and included additional centromere proximal sequences that are missing in the *iab-7*^*R73*^ deletion (Fig 1B). As indicated in the Fig 1B, *F8*^*550*^ extends 265 bp beyond the proximal endpoint of the *F8*^*659*^ and includes the minimal PTS (290 bp) tested in transgenic lines [63]. The male and female cuticle preparations in Fig 2 show that this smaller re-centered fragment fully rescues the *Fab-7*^*attP50*^ deletion. Whereas in *Fab-7*^*attP50*^ males, A6 is transformed into A7 (and thus almost completely disappears), the size, pigmentation and also morphology of the A6 cuticle in *F8*^*550*^ males is like that of wild type flies. The same is true for *F8*^*550*^ females. Instead of a duplicate copy of A7 in *Fab-7*^*attP50*^ females (Fig 2), A6 resembles wild type and its morphology is clearly distinct from the adjacent A7 segment. Thus, like the large *F8*^*659*^ fragment of Iampietro et al. [59], *F8*^*550*^ blocks cross-talk between *iab-6* and *iab-7*. However, it differs from *F8*^*659*^ in that it is also able to support regulatory interactions between *iab-6* and the *Abd-B* promoter and the morphology of the sternites and tergites in A6 (PS11) is wild type.

### *Fab-8* dCTCF recognition sequences are required for blocking, bypass, and domain definition

*Fab-8* has two closely linked binding sites for the conserved insulator protein dCTCF, which are arranged in opposite orientations [64,65]. Reporter assays in flies and tissue culture cells indicate that these two dCTCF sites are important in transgene assays for both enhancer blocking and insulator bypass [54,60,64,66–70]. However, it is not known whether the dCTCF sites are required for *Fab-8* blocking and/or bypass activities in the context of BX-C. To address this question, we introduced a mutant version of the *F8*^*550*^ fragment, *F8*^*550mCTCF*^, which lacks both dCTCF binding sites, into the *Fab-7*^*attP50*^ landing site. The cuticle phenotype of *F8*^*550mCTCF*^ flies points to roles in blocking and bypass.

Like the starting *Fab-7*^*attP50*^ platform, the adult *F8*^*550mCTCF*^ males lack the A6 segment indicating that PS11 is fully transformed into a copy of PS12. A similar result is observed in adult females: *F8*^*550mCTCF*^ females have two nearly identical copies of an A7-like segment (Fig 2). These findings indicate that the dCTCF sites are required for blocking activity. A role in bypass is suggested by the patchy pigmentation of the A5 tergite (PS10) in *F8*^*550mCTCF*^ males (Fig 2). Though the severity of this phenotype is clonally restricted and variable, it is fully penetrant. This effect on A5 pigmentation indicates that the *F8*^*550mCTCF*^ replacement boundary interferes with or fails to fully support regulation of *Abd-B* in PS10 by the *iab-5* domain. It is quite possible that the dCTCF sites in the *Fab-8* replacement are also important for *iab-6<->Abd-B* regulatory interactions. However, since ectopically activated *iab-7* and not *iab-6* regulates *Abd-B* in PS11 (and PS12), in the *F8*^*550mCTCF*^ replacement this possibility cannot be confirmed.

While the A6->A7 transformations in *F8*^*550mCTCF*^ males and females indicates that the dCTCF sites are required to block crosstalk between *iab-6* and *iab-7*, it is important to note that phenotypes are different from mutations that remove only the *Fab-7* boundary. The *Fab-7* boundary deletion mutants display a mixed GOF and LOF transformation of PS11. Exclusively GOF transformations are only observed in *Fab-7* deletions that remove not only the boundary but also the adjacent HS3 *iab-7* PRE (see *Fab-7*^*attP50*^ flies, Fig 2). Remarkably, even though HS3 is included in the *F8*^*550mCTCF*^ replacement, there is no evidence of any LOF (or mixed GOF/LOF) phenotypes in A6. This means that the mutations in the dCTCF bindings sites must have effects on *Abd-B* regulation in PS11 that go beyond a failure to block *iab-6<->iab-7* cross talk.

The phenotypes evident in *F8*^*550mCTCF*^ females support this conclusion. As can be seen in Fig 2, A6 is completely transformed into a duplicate copy of A7. However, in both the duplicate A7 and A7 itself, there are some abnormalities that are not evident in A7 in wild type females. One of these is the bristle pattern on the duplicated A7 sternites. In wild type females, the sternite bristles in A7 all point downwards and most are angled slightly towards the center of the sternite. This same bristle pattern is observed in the duplicated A7 tergites of the *Fab-7*^*attP50*^ deletion (see Fig 2). In contrast, the bristles in the duplicate A7 and the A7 sternites of *F8*^*550mCTCF*^ are rotated nearly 90° so that they point inward. Another difference is in the pigmentation of tergites. In wild type, A6 and A7 (but not A8) are pigmented. The same is true in the *Fab-7*^*attP50*^; both the duplicated A7 and A7 itself are pigmented. This is not the case in *F8*^*550mCTCF*^. Neither of these tergites have pigmentation. These findings suggest that Abd-B expression is abnormal in both of these segments in the *F8*^*550mCTCF*^ mutant.

**Fig 3:**
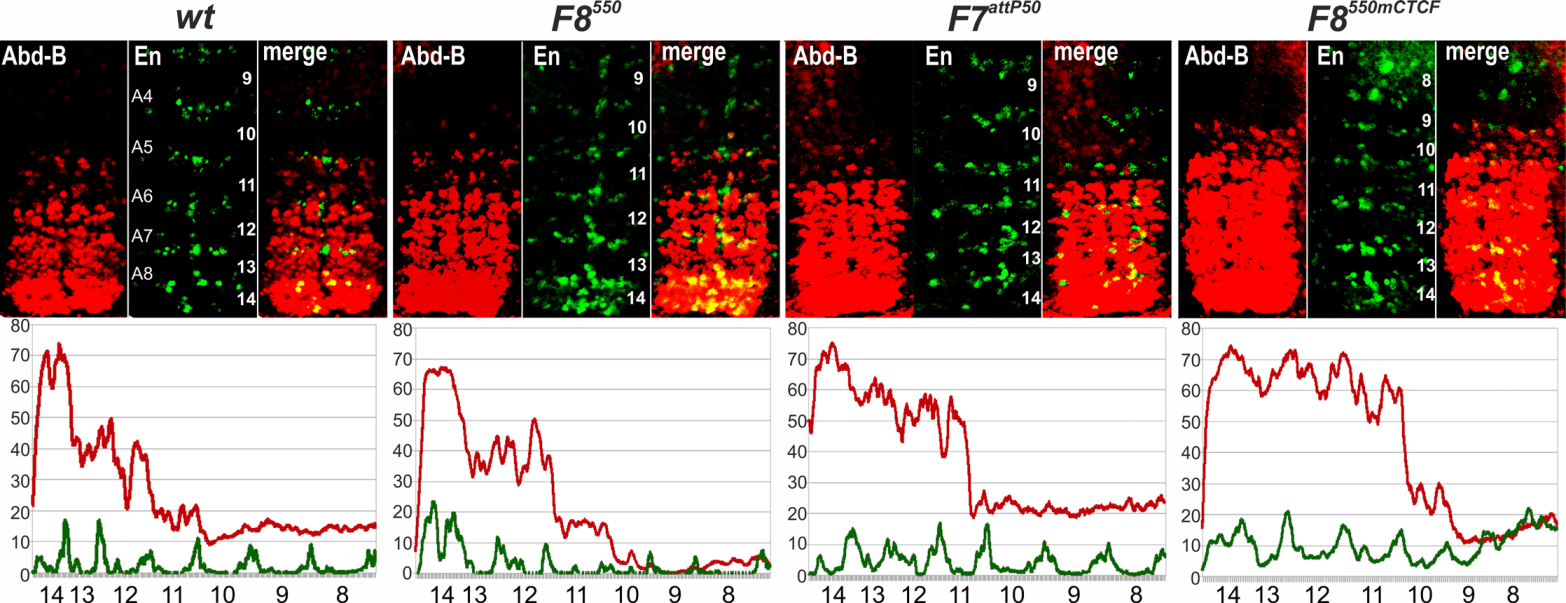
*Abd-B* expression in *Fab-7* replacement embryos. Each panel shows a confocal image of embryonic CNS of stage 14 embryos stained with antibodies to Abd-B (red) and Engrailed (En, green). En is used to mark parasegments, which are numbered from 9 to 14 on the right side of the panels; approximate positions of segments are shown on the left side of the wild type (*wt*) panel and marked A4 to A8 (see Fig 1A for the adult segment numbering). The wild type expression pattern of Abd-B in the embryonic CNS is characterized by a stepwise gradient of increasing protein level from PS10 to PS14. *F8*^*550*^ embryos have intensity and extent of Abd-B expression similar to wild type. *F7*^*attP50*^ embryos have Abd-B expression level in PS11 roughly equal to that in PS12, reflecting *iab-7* activity in PS11. *F8*^*550mCTCF*^ embryos demonstrate an unexpected increase of Abd-B expression in PS11 and 12 so, that it is close to the level of expression seen in PS13. The lower panels show plot profiles of relative fluorescence intensity in the respective images from the upper panels, red lines for Abd-B and green lines for En. Parasegments are numbered from 8 to 14.

To explore the effects of mutating the dCTCF sites further, we examined Abd-B expression in the embryonic CNS. Unexpectedly, two different patterns of expression were observed. The first fits with the exclusively GOF transformation of A6 and may also explain the abnormalities evident in duplicated A7 segments in adult *F8*^*550mCTCF*^ females. In these embryos, high and nearly equal levels of Abd-B expression are observed in PS11, PS12, and PS13 (Fig 3). In the second, the levels of Abd-B expression are also similar in all three parasegments; however instead of resembling that normally seen in PS13, the levels of Abd-B expression in the three segments are relatively low and more like that observed in PS11 or PS12 (S1 Fig**)**. In addition, in some embryos, the levels of Abd-B in a subset of PS12 cells is actually higher than that in PS13 cells (S1 Fig**)**. While clearly abnormal, the second pattern does not fit with the adult cuticle phenotypes. It is possible that the regulatory effects of the mutations in the dCTCF sites differ in the two tissues. In this case, there would be a “choice” between two alternative regulatory states in parasegments PS11-13 in the embryonic CNS, either elevated and nearly PS13-like or reduced and PS11/12-like. Alternatively, the expression pattern in the CNS may evolve from low in PS11-PS13 to high in these parasegments as the embryos develop.

### dCTCF blocks cross talk, but does not support bypass

**Fig 4:**
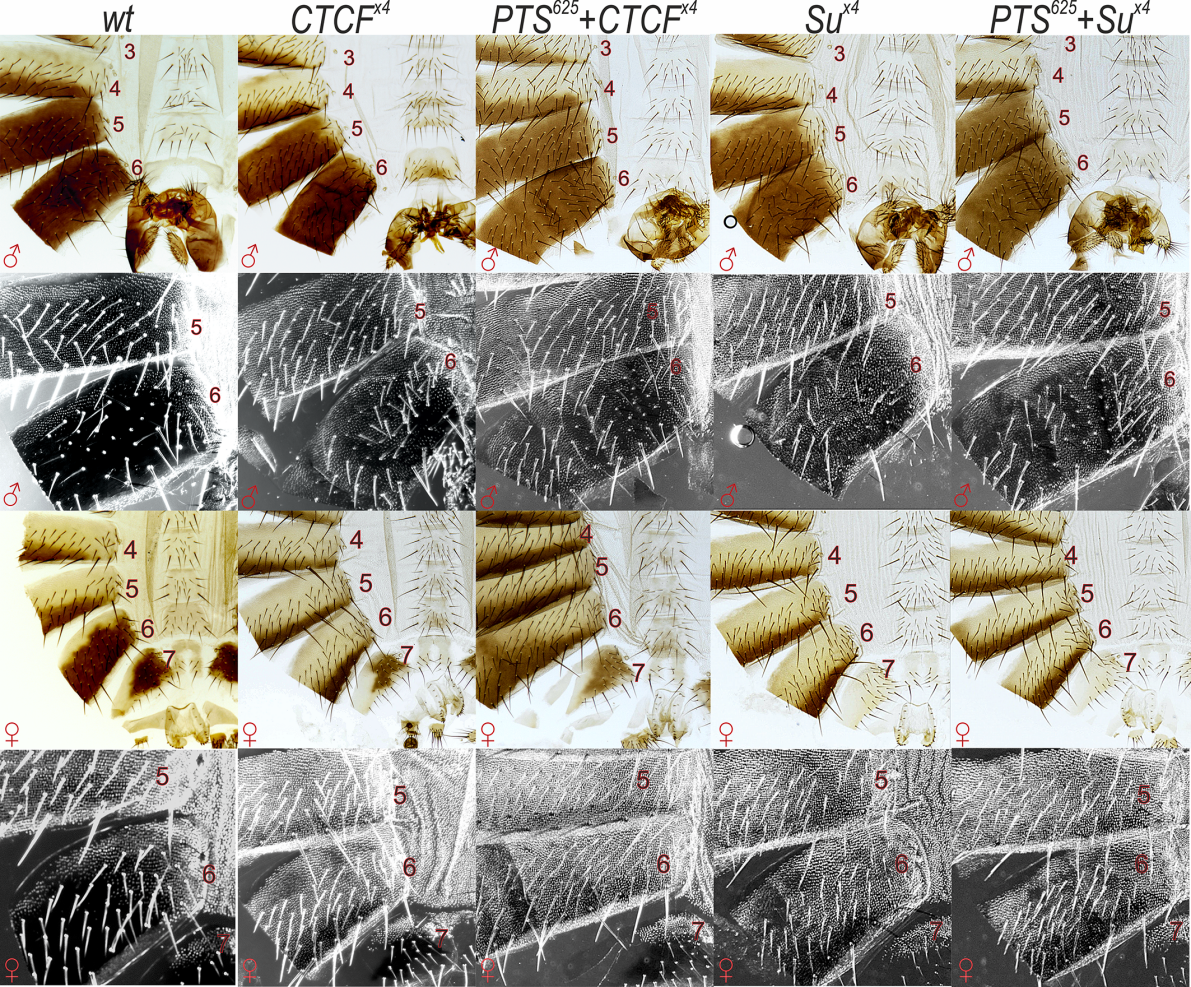
The phenotypic effects of *Fab-7* replacement by dCTCF or Su(Hw) binding sites, with and without PTS. In all shown homozygous mutant males A6 is transformed into A5. The phenotypic effects are the same as in the case of *gypsy* or *scs*^*min*^ swapping. In all shown homozygous mutant females, the transformation of A6 to A5 is evident from the appearance of a uniform trichome pattern on the entire surface of A6 tergite (dark field images).

The phenotypic effects of the *F8*^*550mCTCF*^ replacement indicate that the dCTCF sites are required for blocking and bypass activity. We wondered whether dCTCF alone would also be sufficient for these activities. To test this possibility, we generated a replacement *Fab-7*^*attP50*^ transgene that has four copies of the dCTCF binding site, *CTCF*^*×4*^ (Fig 4). *CTCF*^*×4*^ blocks cross talk between *iab-6* and *iab-7* and there is an A6-like segment in replacement males. However, the A6 segment is not wild type in males or in females. Unlike more anterior sternites, the A6 sternite in wild type males is devoid of bristles and has a horseshoe shape. In *CTCF*^*×4*^ males, the A6 sternite is covered in bristles and the shape is identical to that in A5. Similarly, in *CTCF*^*×4*^ females, the pigmentation of the A6 tergite resembles that of A5 in wild type. An A6->A5 (PS11->PS10) transformation is also evident in the dark field images in Fig 4. In wild type flies, the A6 tergite has two bands of trichomes. One extends along the ventral edge of the tergite, while the other occupies part of the anterior edge. In male and female *CTCF*^*×4*^ flies, the trichomes cover the entire tergite, indicative of an A6->A5 LOF transformation. Though *CTCF*^*×4*^ appears to completely eliminate regulation of *Abd-B* by *iab-6*, the effects on *iab-5* activity are considerably less severe. Fig 4 shows that there is some weak depigmentation of the A5 tergite in males; however, though this LOF phenotype is variable much like that observed for the *F8*^*550mCTCF*^ mutant boundary.

### Sequences deleted in *iab-7*^*R73*^ are important for bypass and proper *Abd-B* regulation in PS12 but not for blocking cross talk

**Fig 5:**
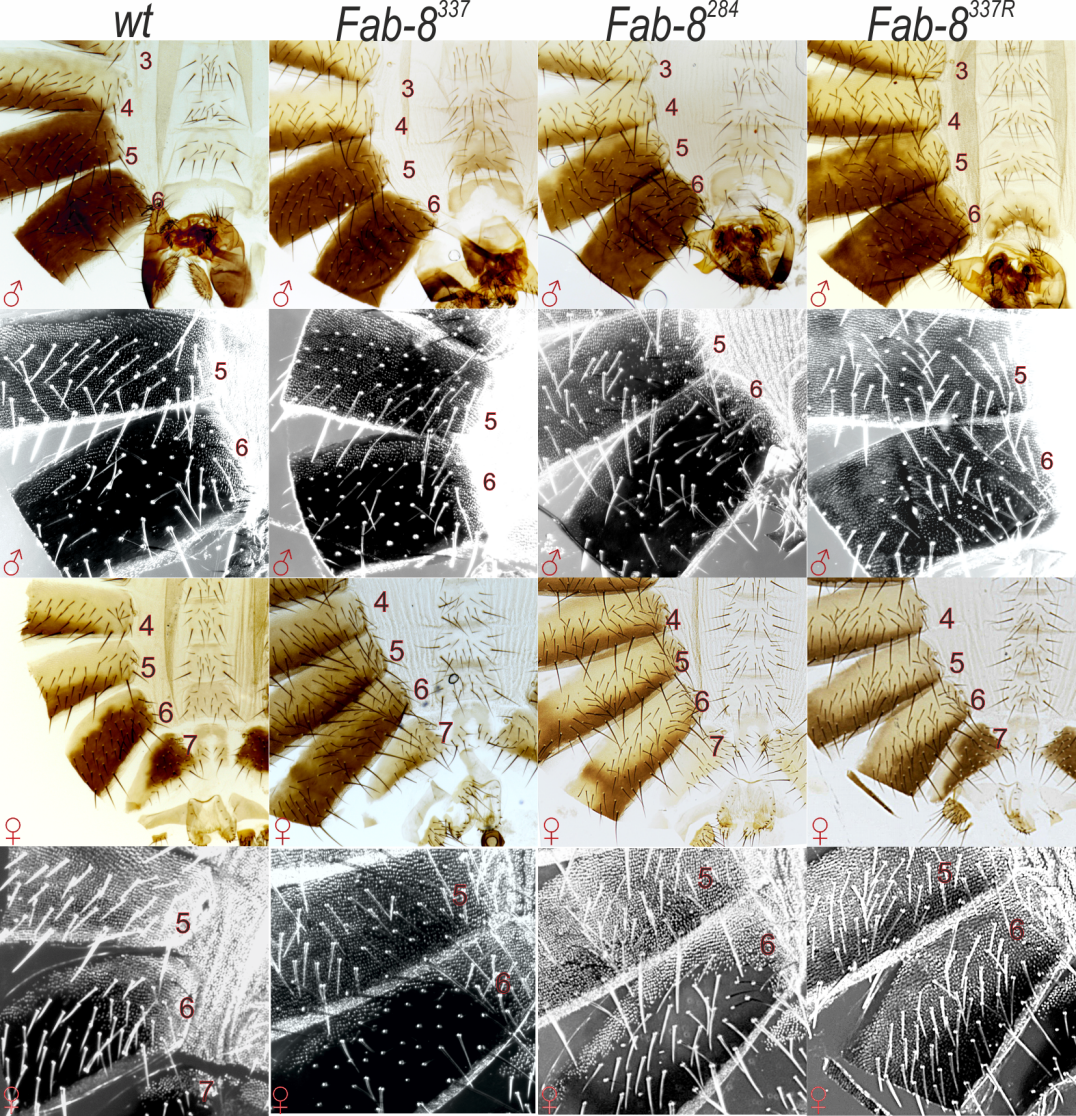
The phenotypic effects of *Fab-7* replacement by *Fab-8*, with part of PTS, and by the *Fab-8* boundary inserted in the reverse orientation. All abdominal segments in *Fab8*^*337*^ males and females have essentially a wild type identity. The removal of the 53 bp of PTS in *Fab8*^*284*^ causes a weak LOF phenotype in A5 and A6. *Fab8*^*337R*^ induces much stronger LOF phenotypes in A6. In males, bristles appear on the A6 sternite and trichomes cover the entire surface of the A6 tergite. There is also depigmenation of the A5 tergite. In females, trichomes cover the entire A6 tergite and the pigmentation pattern resembles that of A5.

**Fig 6:**
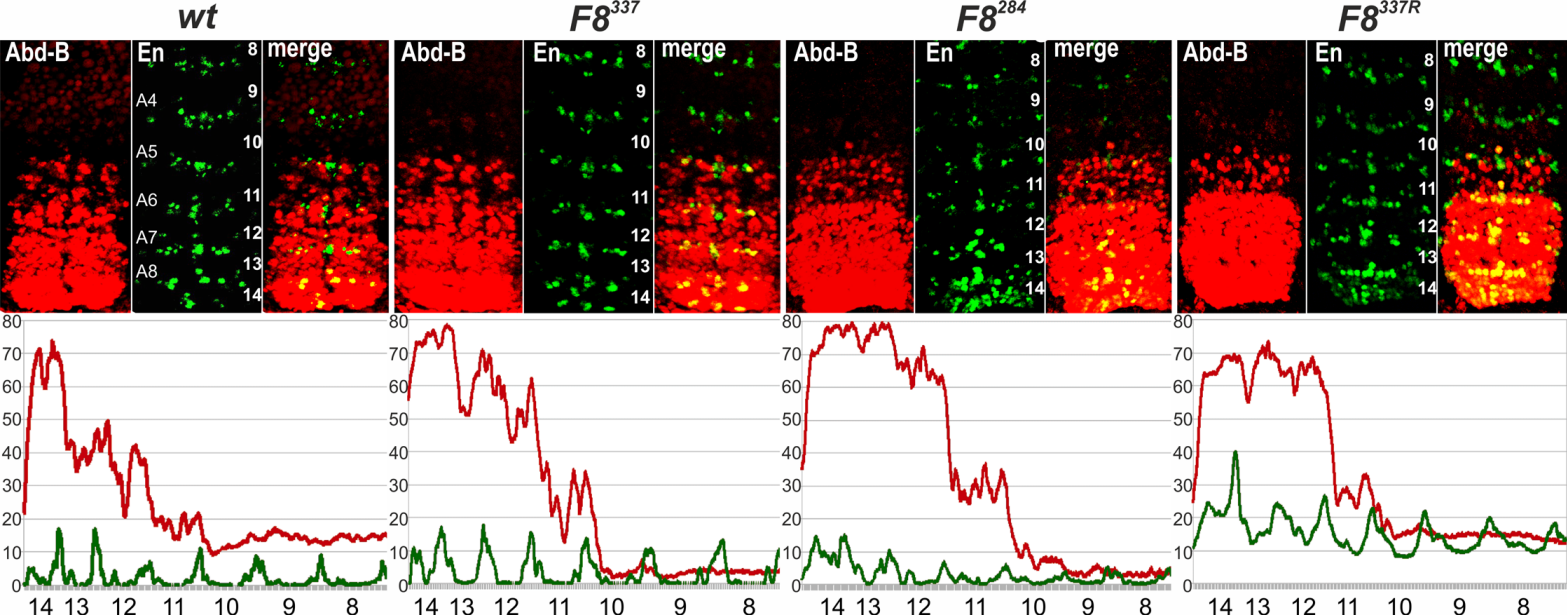
Patterns of Abd-B expression in *CTCF*^*×4*^, *Fab8*^*337*^, *Fab8*^*284*^, and *Fab8*^*337R*^. Embryos were stained and marked as in Fig 3. Like wild type, Abd-B expression in PS10-13 in *F8*^*337*^ embryos increases in a stepwise pattern from one parasegment to another. In *F8*^*284*^ embryos, the level of Abd-B in PS12 is elevated and close to that of PS13. In *F8*^*337R*^, expression levels of Abd-B in PS13 and PS12 are nearly equal, while the Abd-B expression in PS11 is reduced. The lower panels show plot profiles of relative fluorescence intensity in the respective images from the upper panels, red lines for Abd-B and green lines for En. Parasegments are numbered from 8 to 14; approximate positions of segments are shown on the left side of the wild type (*wt*) panel and marked A4 to A8 (see Fig 1A for the adult segment numbering).

The weak *iab-7* LOF phenotype of *iab-7*^*R73*^ would be consistent with the idea that sequences in this 820 bp deletion contribute to insulator bypass. As shown in Fig 1B, the *iab-7*^*R73*^ deletion includes the entire PTS. However, since there is a deletion, *Δ330*^*iab-7*^, that is slightly smaller than *iab-7*^*R73*^, which has the same centromere proximal breakpoint as *iab-7*^*R73*^ but is wild type (Fig 1B), it seems likely that most of these PTS sequences are not needed for *Fab-8* function. Instead, the critical sequences would be located between the centromere distal endpoint of the *Δ330*^*iab-7*^ deletion and the centromere proximal end of the 659 bp fragment used by Iampietro et al. [41,59]. If this is correct, a *Fab-8* fragment (*F8*^*337*^) that includes this sequence but not more centromere proximal sequences, should substitute for *Fab-7*. The *Fab8*^*284*^ boundary is identical to *Fab8*^*337*^, except that the distal endpoint is the same as in Iamperio et al. [59] (Fig 1B). Fig 5 shows that this is the case. The morphological features in segments A5-A8 of the adult cuticles of *F8*^*337*^ males and females are those expected in wild type. The same is true for the pattern of Abd-B expression in the embryonic CNS (Fig 6).

The proximal end-point of *F8*^*337*^ extends 53 bp beyond the proximal endpoint of the *F8*^*659*^ fragment used in the experiments of Iampietro et al. (Fig 1B). To test whether this 53 bp sequence is needed for *Fab-8* function, we generated a deletion replacement, *F8*^*284*^. Like *F8*^*337*^, the smaller *F8*^*284*^ boundary blocks crosstalk between *iab-6* and *iab-7*, and there is no evidence of GOF transformation in A6 (PS11). On the other hand, unlike *F8*^*337*^, the morphological features of *F8*^*284*^ adults are abnormal and there are fully penetrant weak LOF phenotypes in both A5 and A6 (Fig 5). In males the A5 tergite has small regions that are depigmented, while in females the pigmentation pattern in A6 often resembles that seen in A5. Normally the A6 sternite in males is devoid of bristles; however, as illustrated in Fig 5, this not the case in the *F8*^*284*^ replacement. In addition, while the shape of the hard cuticle of the A6 sternite in the male fly shown in the figure resembles wild type, in other males the A6 sternite has a shape much more similar to that in A5. Finally, in a subset of *F8*^*284*^ male and female flies, we observed small clones of trichomes in the posterior and dorsal regions of the A6 tergite that are normally devoid of trichomes.

### The *Fab-8* PTS sequence does not rescue the bypass defects of dCTCF and Su(Hw) multimers

Even though the dCTCF sites in *Fab-8* contribute to both blocking and bypass, multimerized dCTCF binding sites alone have blocking activity but do not in themselves support bypass. In the case of the dCTCF sites in *Fab-8*, it seems likely from our deletion analysis that *cis*-acting elements in the *F8*^*337*^ replacement in addition to the 53 bp sequence from the distal end of the PTS contribute to bypass activity. For this reason, we did not expect this 53 bp PTS sequence to complement the bypass defects of the multimerized dCTCF binding sites. On the other hand, since the full length 625 bp *Fab-8* PTS is able, on its own, to mediate enhancer bypass of a heterologous *su(Hw)* insulator in transgene assays, we wondered whether the full PTS element would be able to rescue the bypass defect of the multimerized dCTCF sites. To test this possibility, we combined the 625 bp PTS with the *CTCF*^*×4*^. Contrary to our expectations, *PTS*^*625*^*+CTCF*^*×4*^ males and females had the same spectrum of LOF phenotypes in A6 and A5 as their *CTCF*^*×4*^ counterparts (Fig 1B and Fig 4).

In previous studies, the bypass activity of the *Fab-8* PTS was tested in combination with the *gypsy* insulator which contains multiple binding sites for the Su(Hw) protein [56]. Thus, a plausible explanation for the failure to rescue the bypass defects of the *CTCF*^*×4*^ replacement is that this PTS functions best in conjunction with *su(Hw)* insulators. To test this hypothesis, we asked whether the same *Fab-8* PTS fragment facilitates bypass of a multimerized Su(Hw) binding sites (*Su*^*×4*^). Like *CTCF*^*×4*^, the multimerized *Su*^*×4*^ replacement blocks cross-talk between *iab-6* and *iab-7*, but fails to support bypass (Fig 4). Moreover, this bypass defect is not rescued by the *Fab-8* PTS (see *PTS*^*625*^+*Su*^*×4*^ in Fig 4), and the same spectrum of LOF phenotypes are observed in A6 and A5 as those seen with *Su*^*×4*^ alone. Taken together with *iab-7*^*R73*^ deletion and the fact that *F8*^*337*^ has full boundary activity, these findings would suggest that the PTS does not function in the same way in the context of BX-C as it does in transgene assays.

### Fab-8 boundary function is orientation dependent

In insulator bypass experiments, *Fab-8* interactions with itself and with other insulators are orientation dependent [30,60]. With only a few exceptions (*Fab-7*: see below), this is a characteristic property of fly insulators in this transgenic assay. Self-interactions are head-to-head, while heterologous interactions can be either head-to-tail or head-to-head. In the case of the BX-C boundaries that define the *Abd-B* domain (Fig 1A), heterologous interactions occur head-to-head. For productive regulatory interactions in the transgenic bypass assay, these BX-C insulators are inserted in opposite orientations (forward<->reverse), so that head-to-head pairing interactions generate a stem loop. However, it is not known whether their relative orientation is important for proper insulator function in the context of BX-C. To explore this issue, we tested whether the orientation of *Fab-8* in BX-C affects the ability of this insulator to rescue the *Fab-7*^*attP50*^ deletion. For this purpose, we introduced the 337 bp *Fab-8* fragment into *Fab-7*^*attP50*^ in the reverse orientation (*F8*^*337R*^).

While this 337 bp *Fab-8* fragment fully rescues the *Fab-7* deletion when it is in the same “forward” orientation as the endogenous *Fab-8* insulator, this is not true when its orientation is reversed (Fig 1B). The effects of inverting the insulator on its activity in BX-C are instructive. As can be seen in Fig 5, *F8*^*337R*^ blocks cross talk between *iab-6* and *iab-7*, and the GOF transformation of PS11->PS12 in male and female *Fab-7*^*attP50*^ adults is completely suppressed. This finding indicates that blocking activity, at least in this particular context, does not depend upon insulator orientation. On the other hand, orientation is critical for insulator bypass, particularly for the *iab-6* regulatory domain. The A6 tergites of both sexes are covered in trichomes—a morphological feature that is found in wild type in A5 but not A6. Also the pigmentation of the A6 tergite in *F8*^*337R*^ females is largely restricted to the posterior edge much like that normally seen in A5. A similar A6->A5 (PS11->PS10) transformation is evident in the A6 sternite of *F8*^*337R*^ males. The sternite has bristles and is shaped like the A5 sternite. The effects on *iab-5* regulation of *Abd-B* are less severe. There is a variable depigmentation of A5 indicative of a PS10->PS9 transformation. Consistent with the LOF phenotypes evident in the adult cuticle, the difference in the levels of Abd-B protein accumulation in PS12 and PS11 in the CNS is greater than normal in *F8*^*337R*^ embryos (Fig 6). Also, the level of Abd-B protein in PS12 compared to PS13 appears to be somewhat elevated.

### *Fab-8* bypass activity does not depend upon the orientation of the *Fab-8* dCTCF sites

Recently, several studies showed that the relative orientation of CTCF sites in mammalian boundary elements is critical for proper insulator function [71–75]. Since the blocking and bypass activity of the *Fab-8* boundary requires the two dCTCF sites, an obvious question is whether either of these functions is connected to their relative orientation within the *Fab-8* boundary. To test this possibility, we changed orientation of one (*F8*^*CTCF Dir-Dir*^) or both of the *Fab-8* dCTCF sites (*F8*^*CTCF Dir-Rev*^) in the *F8*^*337*^ replacement (S2 Fig). The morphological features in segments A5-A8 of the adult cuticles prepared from *F8*^*CTCF Dir-Dir*^ and *F8*^*CTCF Dir-Rev*^ adult flies resembles that expected for the wild type (S3 Fig). Thus, the relative orientation of dCTCF sites does not seem to be critical for either blocking or bypass activity of the *Fab-8* replacement boundary (Fig 1B).

### Functioning of Fab-7 in BX-C is orientation independent

**Fig 7:**
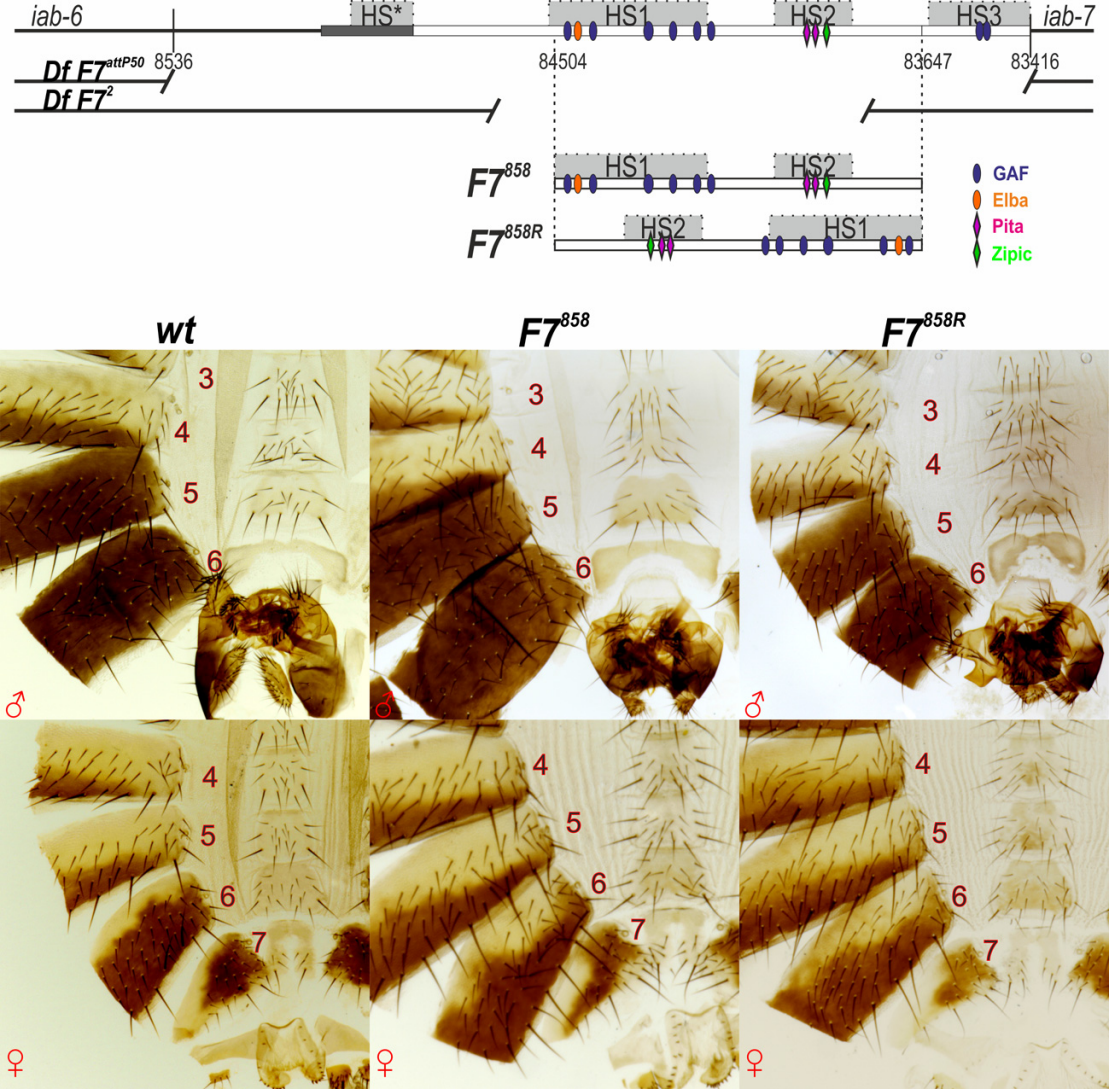
An effect of *Fab-7* orientation on the *Abd-B* expression. Molecular maps of the *iab6*-*iab7* region, and *Fab-7* boundary replacement fragments. The *Fab*-7 insulator is represented by wide white bar on the molecular coordinate line. The part of PTS-6 is marked by dark gray. *Fab-7* has four DNase hypersensitive sites (HS*, HS1-3) shown as light gray boxes. Two variants of *Fab-7* deletions are indicated by gaps in black lines. In our experiments, *F7*^*858*^ and *F7*^*858R*^ fragments were inserted in *F7*^*attP50*^, with a restored HS3 *iab-7* PRE in both cases. Known protein binding sites are indicated with colored ovals and rhombi. Binding factors, common with Fab-8, are shown as ovals: blue–GAF, orange–Elba/Insv. The non-common factors–as rhombi: rose–Pita, green–Zipic. Cuticles of *F7*^*858*^ and *F7*^*858R*^ males and females look essentially as wild type.

As mentioned above, *Fab-7* differs from *Fab-8* and most other fly insulators in that its bypass activity in transgene assays is orientation independent [76]. Since reversing the orientation of the *Fab-8* insulator disrupted its ability to replace *Fab-7*, we wondered whether orientation was important for *Fab-7* in its endogenous context. To answer this question, we inserted two different versions of an 858 bp fragment that contains the two major nuclease hypersensitive sites, HS1 and HS2, that are associated with the *Fab-7* boundary, next to the *iab-7* PRE hypersensitive site HS3. In one version, *Fab-7*^*858*^, the sequences spanning the two major Fab-7 hypersensitive sites, HS1+HS2, were in the same orientation as they are in the endogenous locus. In the other, *Fab-7*^*858R*^, the HS1+HS2 sequences are in the opposite orientation. The cuticle preps in Fig 7 show that both versions of the 858 bp fragment fully rescue the *Fab-7*^*attP50*^ deletion. Thus, as was observed in the transgene bypass assay, *Fab-7* function in BX-C is orientation independent.

## Discussion

In the studies reported here we have used a gene replacement strategy to study the properties of *Fab-8* that enable it to function in BX-C (see summary table in Fig 1B). We show that a minimal fragment spanning the *Fab-8* nuclease hypersensitive site and including the distal part of PTS sequences fully rescues a *Fab-7* deletion. It blocks crosstalk between *iab-6* and *iab-7*. It is also permissive for interactions between the downstream *iab-5* and *iab-6* regulatory domains and the *Abd-B* promoter. The CTCF protein is well known because of its ability to block enhancer-promoter interactions and is found in many insulators from insects to vertebrates [77,78]. Transgene experiments have shown that mutations in the two *Fab-8* dCTCF binding sites compromise its enhancer blocking activity [63,64,69,73,75–77]. The same mutations completely disrupt the ability of the *Fab-8* replacement to block crosstalk between the *iab-6* and *iab-7* regulatory domains.

Conversely, when dCTCF sites are multimerized, they are sufficient to prevent crosstalk between *iab-6* and *iab-7*. On the other hand, the multimerized binding sites do not substitute for *Fab-7*, because in this context they lack bypass activity and block the *iab-6* (and to a lesser extent *iab-5*) regulatory domain from regulating *Abd-B*.

This is not the only link between generic boundary functions and the ability to replace *Fab-7*. In bypass assays, the dCTCF sites are required for orientation self-pairing between *Fab-8* boundaries and for heterologous interactions with other nearby BX-C boundaries. In addition, a sequence at the distal end of the PTS is required for specific interactions with *Fab-7*, *Fab-8*^*mCTCF*^, and *AB-I* [30,60]. While self-interactions between *Fab-8* boundaries in *cis* do not occur in wild type flies, in our experimental design, self-interactions between the *Fab*-8 boundary in its normal location and the *Fab-8* replacement are expected. As would be predicted from previous transgene bypass experiments, mutations in the dCTCF binding sites and deletion of the PTS, interfere with *Abd-B* regulation by the downstream regulatory domains. For the PTS deletion, *Abd-B* regulation by both *iab-5* and *iab-6* is partially compromised. In the case of the dCTCF sites, these effects can only be seen for *iab-5* (PS10), because *iab-7*, not *iab-6*, regulates *Abd-B* in PS11. In this context, it is also important to note that the only part of the 625 bp PTS that is needed for full bypass activity is an 83 bp sequence at its very distal end, while the remainder of the PTS is completely dispensable. Moreover, even when all but 30 bp of the 625 bp PTS is deleted (*F8*^*284*^), the effects on *iab-6* and *iab-5* regulatory activity are quite modest compared, for example, to that seen for either *CTCF*^*×4*^ or *F8*^*337R*^. This would be consistent with the rather weak LOF phenotypes of the *Fab-8*^*R73*^ deletion, and argues that the PTS by itself, does not have an essential role in the bypass activity of the *Fab-8* boundary in the context of BX-C. Since multimerized dCTCF sites lack bypass activity, it seems likely that *cis*-acting elements contained within the smaller *F8*^*284*^ substitution will turn out to be critical for bypass activity. Of course, though our deletion experiments argue that the PTS makes at most only a minimal contribution to *Fab-8* bypass activity, our experimental design does not exclude a scenario in which the PTS is redundant with the bypass elements in *F8*^*284*^. However, arguing against this scenario is the fact that the full 650 bp PTS fails to complement the bypass defects of not only *CTCF*^*×4*^ but also *Su*^*×4*^. Since the PTS is able to mediate bypass of a *gypsy* element (which contains 12 Su(Hw) binding sites) in a transgene assay, it seems possible that its activity is entirely context dependent—in this case, the specific identity, combination and arrangement of enhancers, insulators, and reporters in the different transgene constructs [56,63].

Yet another connection between the bypass activity of BX-C insulators in transgene assays and bypass in BX-C, is orientation dependence. The bypass activity of *Fab-8* in transgene assays differs depending on insulator orientation [30,60]. The same is true in our replacement experiments. *Fab-8* substitutes for *Fab-7* when it is inserted in the same relative orientation in BX-C as the endogenous *Fab-8* boundary. On the other hand, when the orientation of *Fab-8* replacement boundary is reversed, it no longer supports bypass (though it still blocks crosstalk between *iab-6* and *iab-7*). Instead, it disrupts interactions between *iab-6* and the *Abd-B* gene much like that observed when *Fab-7* is replaced by the completely heterologous insulators *su(Hw)* and *scs* [58]. Notably, however, the effects of *su(Hw)* and *scs* on *Abd-B* regulation by *iab-5* and *iab-6* are orientation independent. Further support for the idea that a bypass type mechanism may be responsible for enabling downstream regulatory domains to skip over one or more boundary elements comes from experiments in which we replaced *Fab-7* with a *Fab-7* fragment. In transgene experiments, *Fab-7* is unusual in that its bypass activity either in combination with itself or with other BX-C insulators is orientation independent [76]. This is also true for the bypass activity of *Fab-7* in BX-C.

**Fig 8:**
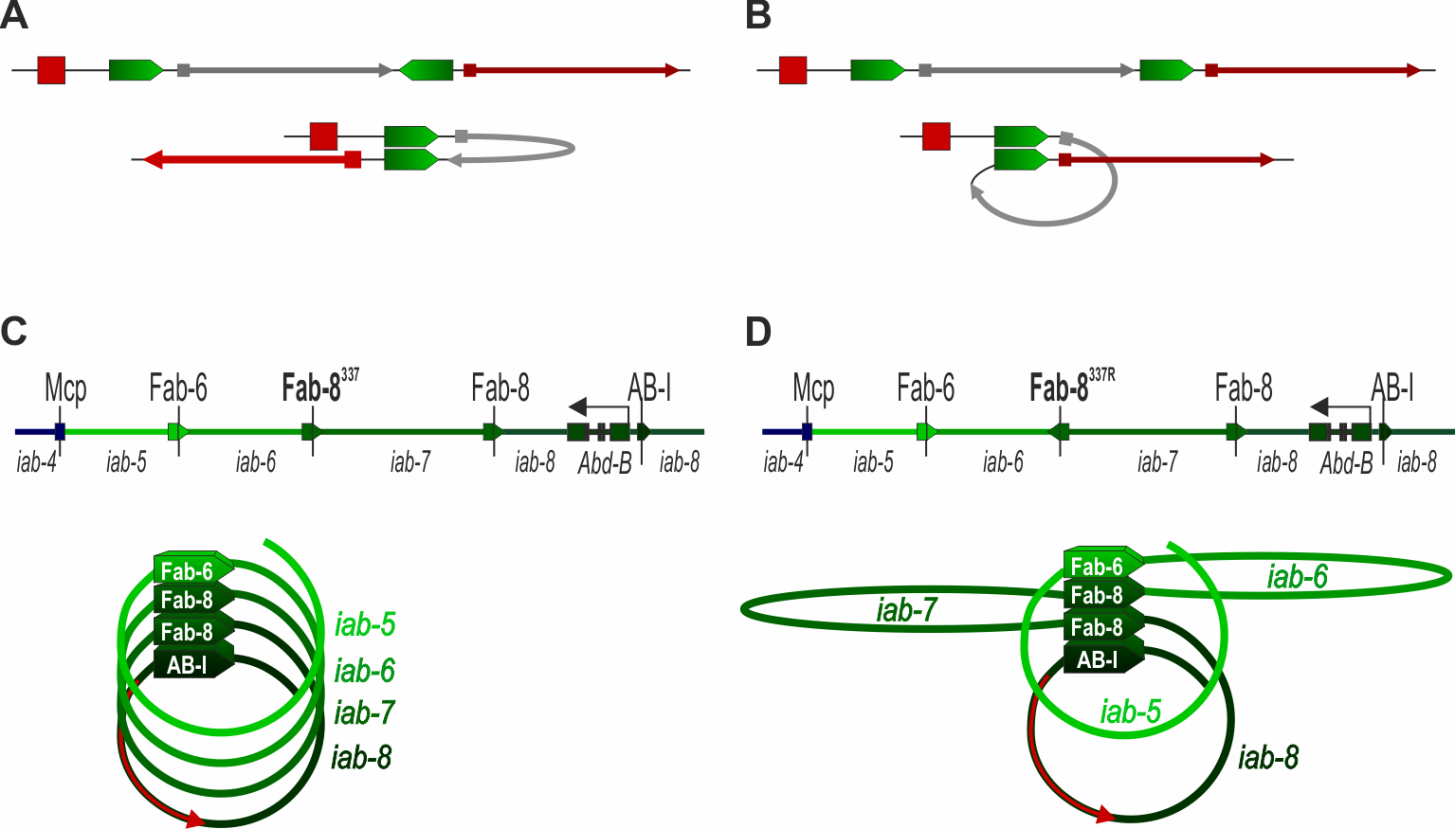
The effect of the relative orientation of insulators on the interaction of *cis*-regulatory elements. In (A) and (B) the insulators pair with each other head-to-head. (A) In the insulator bypass transgene assay, a stem-loop is formed when the two insulators are in opposite orientation. This configuration is favorable for communication between regulatory elements located outside of the stem-loop. (B) When the insulators in the transgene are in the same orientation, pairing leads to the formation of a circle-loop that spatially separates regulatory elements. (C, D) The effect *F8*^*337*^ orientation on formation of chromatin loops. *Abd-B* regulatory region is shown at the top as green lines of different shades, with dark reflecting higher level of Abd-B expression. Insulators are shown as pentagon arrows, that indicate orientation of Fabs, with the same color as the *iab* domains they delimit. (C) In *F8*^*337*^, *Fab-8* insulators are in the same orientation and head-to-head pairing between them would lead to the formation of a series of circle-loops. In this illustration the circle-loops are wound (arbitrarily) in a clockwise direction giving a right-handed helix. (D) The reversal of Fab-8^337^ insulator in *F8*^*337R*^ disrupts this helical structure and introduces two stem-loops. These loops correspond to *iab-6* and *iab-7*.

While these similarities argue that some type of bypass mechanism is likely involved in skipping over intervening boundary elements in the *Abd-B* region of BX-C, there is an important difference between bypass in BX-C and bypass in transgene assays. As illustrated in Fig 8A, BX-C insulators pair with each other head-to-head. When they are in an opposite orientation in the transgene, head-to-head pairing generates a stem-loop structure that brings the enhancer in close proximity to the reporter. By contrast, in transgene assays, head-to-head pairing of insulators that are in the same relative orientation, as they are in BX-C, generates a “circle-loop” and this topological configuration is not favorable for contacts between the enhancer and promoter flanking the paired insulators (Fig 8B).

In the BX-C *Abd-B* domain, all of the insulators are oriented in the same (by convention “forward”) direction with respect to each other. They are also predicted to pair with each other head-to-head [60]. If each insulator interacts with its flanking neighbors, the predicted topology of the entire domain, when the *Fab-8* (*F8*^*337*^) replacement is in the “forward” orientation (same as the endogenous *Fab-8*), would be a series of “circle-loops” linked together at their base by interacting insulators (Fig 8C) [29,30,52,79,80]. In the illustration in Fig 8C, all of the circle-loops are wound in a clockwise direction, giving a right-handed helix.

While the actual *in vivo* configuration of the loops comprising the *Abd-B* regulatory domains cannot be determined with techniques currently available, it is clear that this organization will be disrupted when the *Fab-8* replacement is in the “reverse” orientation. As illustrated in Fig 8D, the introduction of the *Fab-8* boundary in the reverse orientation (*F8*^*337R*^) would disrupt the helical arrangement of *Abd-B* regulatory domains. Head-to-head pairing between *Fab-6* and *F8*^*337R*^ and between *F8*^*337R*^ and *Fab-8* generates stem-loops, not circle loops. The first stem-loop corresponds to the *iab-6* regulatory domain, while the second corresponds to *iab-7*. In this stem-loop configuration, *iab-6* and the *Abd-B* transcription unit are on opposite sides of the insulator complex and contacts between regulatory elements in *iab-6* and the *Abd-B* promoter would be disfavored. This would dovetail with the strong LOF A6->A5 transformation observed in *F8*^*337R*^ flies, and the reduced Abd-B expression evident in embryos. Importantly, the two stem-loops formed by head-to-head pairing of *F8*^*337R*^ would also disrupt other possible configurations of the circle-loops and interfere with *Abd-B* regulation by *iab-6*. Since the spatial relationship between the *iab-5* and *Abd-B* circles would remain largely the same, one might expect that the effects of the reversed boundary on *iab-5* activity would be less severe than those observed for *iab-6*.

It is interesting to note that equivalent orientation dependent alterations in the regulatory interactions have been observed in mammals when the relative orientation of neighboring CTCF sites is flipped. For this reason, it was somewhat surprising to find that altering the relative orientation of the two dCTCF sites in the *Fab-8* has no apparent effect on the activity of the replacement boundary. The replacements still block *iab-6⇓◇iab-7* cross talk and fully support bypass. This finding indicates that the orientation dependence of the *Fab-8* replacement must be largely dictated by the asymmetric binding of other, unknown factors to the sequences within the minimal *F8*^*337*^ boundary. Thus, though it seems likely that dCTCF sites contribute to the orientation dependence, altering their orientation is not sufficient to override the activity of the other factors.

Unlike *Fab-8*, *Fab-7* pairing interactions with itself and with its neighbors are orientation independent. This means that *Fab-7* pairing with neighboring insulators could generate circles, stem-loops or both. As changing the orientation of the *Fab-7* insulator had no effect apparent on boundary function, a reasonable speculation at this point is that its pairing interactions are dictated by the orientation dependence of the neighboring boundaries and consequently, that it participates in circle (Fig 8C), not stem-loop formation (Fig 8D).

Taken together our model suggests that directional interactions between boundaries in BX-C are essential for the proper spatial organization of the *iab* enhancers relative to the *Abd-B* promoter. Likely, this is not in itself sufficient to generate productive regulatory interactions between the appropriate *iab* enhancers and the *Abd-B* promoter in each parasegment. Instead, additional elements might be needed. One such element is the promoter tethering element or PTE. Studies by Drewell and colleagues [81–83] have identified a PTE located in between the *Abd-B* transcription start site and the insulator-like element *AB-I*. They have shown that PTE can mediate productive contacts between the *iab-5* enhancer and the *Abd-B* promoter in transgene assays. In this case, insulator-insulator interactions would function to organize the *iab* enhancers into the appropriate three-dimensional loop configuration, while direct contact between the enhancers and the *Abd-B* promoter would be dependent on PTE-enhancer interactions. In this context, it is interesting to note that PTEs were also found in promoters of several other genes including *Scr* [84,85], *white* [86], *yellow* [87], *even skiped* [88], and *engrailed* [89].

## Materials and Methods

### Generation of the *Fab-7*^*attP50*^ integration platform

The strategy to create the *Fab-7*^*attP50*^ landing platform is diagrammed in S4 Fig in the Supporting Information and described in detail in [71]. Fragments *F8*^*550*^ (64038–64587), *F8*^*337*^ (64038–64375), *F8*^*284*^ (64038–64322), *PTS*^*625*^ (64292–94916) and *F7*^*858*^ (83647–84504) were obtained by PCR amplification and sequenced. The coordinates are given according to the published sequences of the Bithorax complex [90]. The *CTCF*^*×4*^ and *Su*^*×4*^ are described in [29].

### Cuticle preparations and antibody staining

Adult abdominal cuticles of homozygous eclosed 3–4 day old flies were prepared essentially as described in Mihaly et al. [40] and mounted in Hoyer's solution. Embryos were stained following standard protocols. Primary antibodies were mouse monoclonal anti-Abd-B at 1:60 dilution (1A2E9, generated by S.Celniker, deposited to the Developmental Studies Hybridoma Bank) and polyclonal rabbit anti-Engrailed at 1:500 dilution (kindly provided to us by Judith Kassis). Secondary antibodies were goat anti-mouse Alexa Fluor 555 and anti-rabbit Alexa Fluor 647 (Molecular Probes). Stained embryos were mounted in the following solution: 23% glycerol, 10% Mowion 4–88, 0.1M Tris-HCl pH 8.3. Images were acquired on Leica TCS SP-2 confocal microscope and processed using Photoshop, ImageJ, Excell, and Calc (LibreOffice) software.

## Supporting Information

S1 Fig Abd-B expression in the embryonic CNS of *F8*^*550mCTCF*^ embryos. Three representative *F8*^*550mCTCF*^ embryos, in which the level of Abd-B expression in the CNS is reduced, are shown. Abd-B protein expression (red, top row), Engrailed (green, middle row), and plot profiles of relative fluorescence intensity in the respective images from the upper panels, red lines for Abd-B and green lines for En (bottom row). Parasegments are numbered from 8 to 14.
(TIF)

S2 Fig An effect of the CTCF binding site orientation in *Fab-8* on the *Abd-B* expression. Molecular map of the *F8*^*337*^ insulator with native and inverted dCTCF binding sites. dCTCF binding sites are shown as red triangles indicating orientation of the sites. The orientation of the dCTCF binding sites in endogenous F8 are reverse–direct. dCTCF binding sites with the changed orientation are marked by yellow border. Cuticles of *F8*^*CTCF Dir-Dir*^ and *F8*^*CTCF Dir-Rev*^ males and females look essentially as wild type.
(TIF)

S3 Fig Sequence of *F8*^*CTCF Dir-Rev*^ and *F8*^*CTCF Dir-Dir*^. The molecular map of the *F8*^*337*^ insulator and *F8*^*CTCF Dir-Rev*^ and *F8*^*CTCF Dir-Dir*^ is the same as in S2 Fig. The PTS sequence is highlighted with gray. Inverted dCTCF binding sites are highlighted with yellow. Elba binding sites are in orange, GAF–in blue, dCTCF–in red.
(PDF)

S4 Fig The strategy to create *Fab-7* replacement lines. On the top: schematic representation of regulatory region of the *Abd-B* gene (green). The 1950 bp Fab-7 region that was deleted in *F7*^*attB50*^ is shown in detail. The hypersensitive sites “*”, HS1, and HS2 are shown as gray boxes. HS3, which comprises the *iab-7* PRE, is shown in blue. *F7*^*attB50*^ landing platform (shown below) contains an *attP* site for the integration of the tested constructs; *lox* and *frt* sites were used for excision of the plasmid body and of *rosy* maker gene. The plasmid that was injected into *Fab-7*^*attp50*^ line, contains *attB* site for integration, HS3 *iab-7* PRE for restoring functional integrity of the *iab-7* domain, *frt* sites for excision of *rosy* gene, *rosy* gene, *lox* sites for excision of the plasmid body (shown below). Testing elements were inserted just in front of *iab-7* PRE. After integration of the plasmid within *Fab-7*^*attp50*^, *ry+* transformants were selected. Then, *rosy* and plasmid cassette were excised by FLP-recombinase, to remove an about 10.2 kb additional sequence between the tested element and *iab-7* in *ry+* line.
(TIF)
