# Supplementary figures and images for: Functional Dissection of the Blocking and Bypass Activities of the Fab-8 Boundary in the Drosophila Bithorax Complex
Source: PLoS Genet. 2016 Jul 18;12(7):e1006188. doi: 10.1371/journal.pgen.1006188 (PMC4948906; doi:10.1371/journal.pgen.1006188)

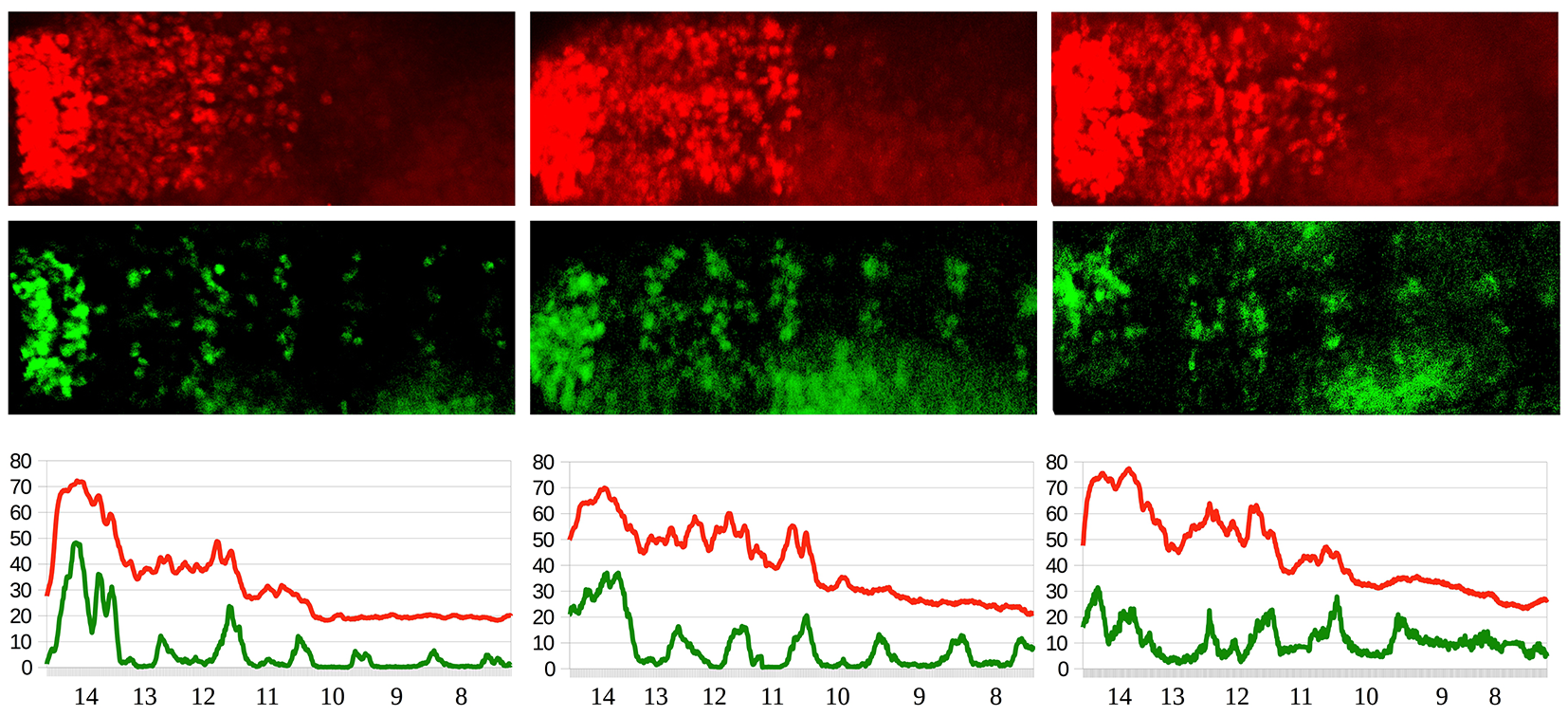

Supplement: S1 Fig — Three representative F8550mCTCF embryos, in which the level of Abd-B expression in the CNS is reduced, are shown. Abd-B protein expression (red, top row), Engrailed (green, middle row), and plot profiles of relative fluorescence intensity in the respective images from the upper panels, red lines for Abd-B and green lines for En (bottom row). Parasegments are numbered from 8 to 14. (TIF) [file pgen.1006188.s001.tif]

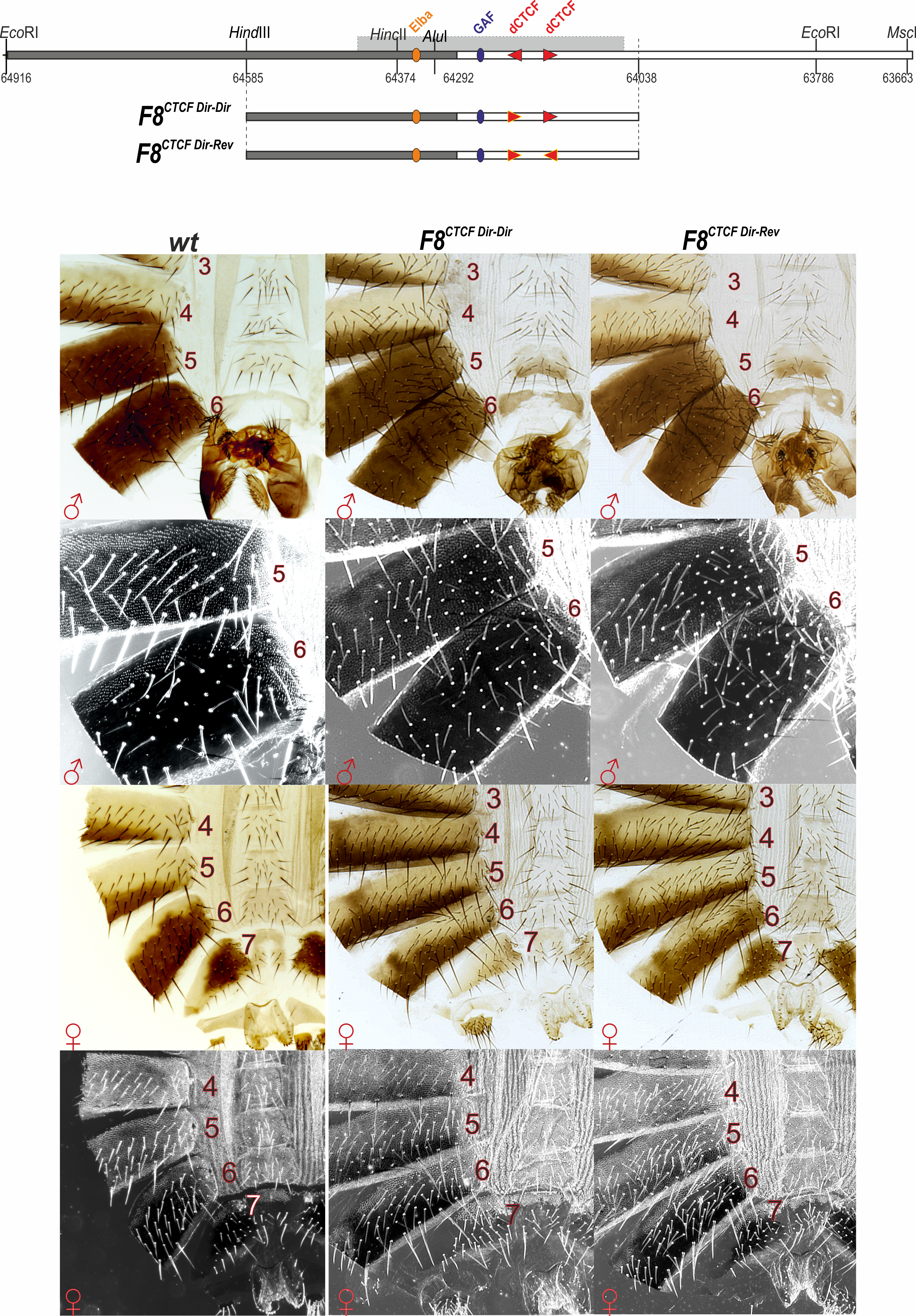

Supplement: S2 Fig — Molecular map of the F8337 insulator with native and inverted dCTCF binding sites. dCTCF binding sites are shown as red triangles indicating orientation of the sites. The orientation of the dCTCF binding sites in endogenous F8 are reverse–direct. dCTCF binding sites with the changed orientation are marked by yellow border. Cuticles of F8CTCF Dir-Dir and F8CTCF Dir-Rev males and females look essentially as wild type. (TIF) [file pgen.1006188.s002.tif]

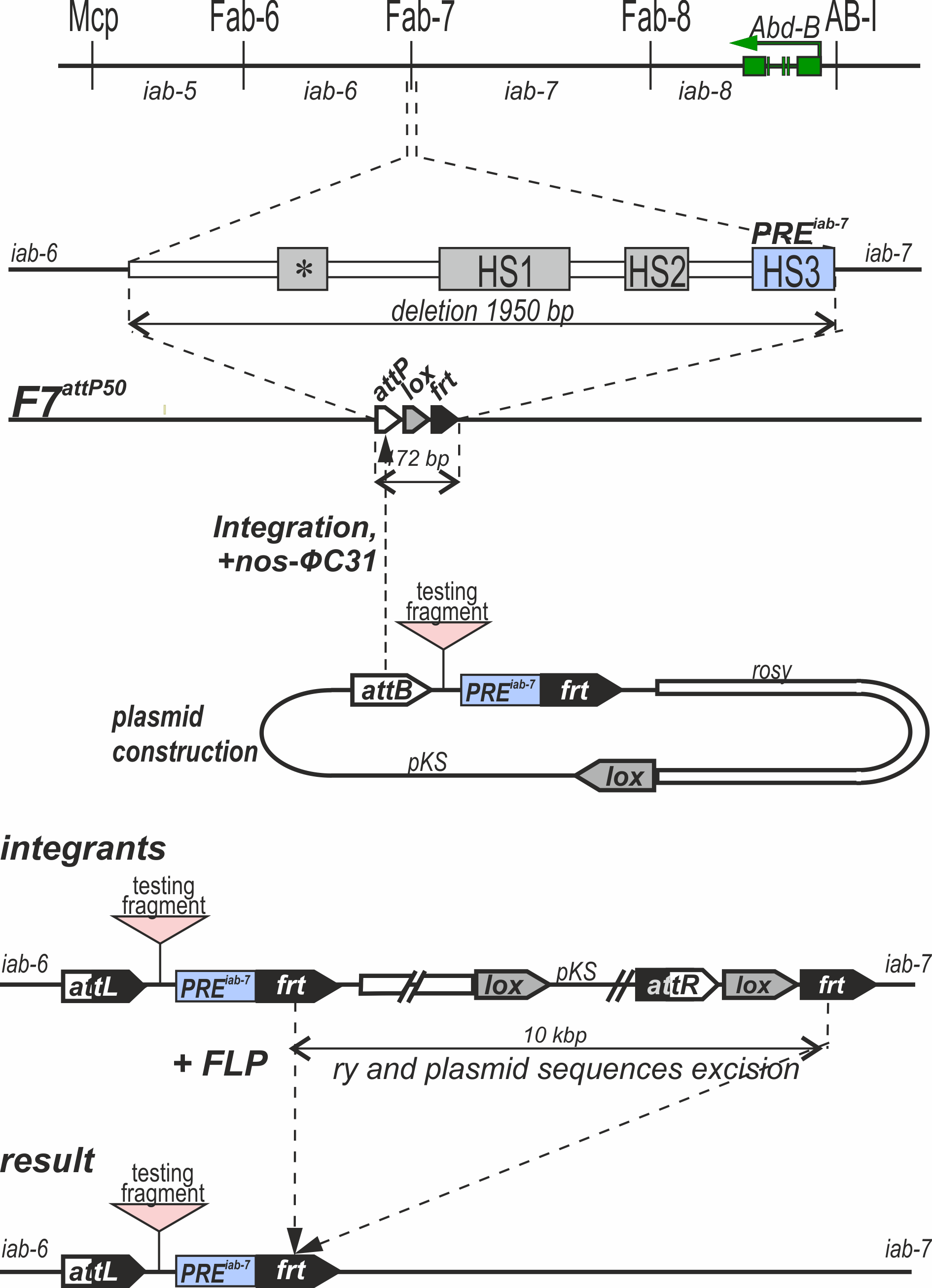

Supplement: S4 Fig — On the top: schematic representation of regulatory region of the Abd-B gene (green). The 1950 bp Fab-7 region that was deleted in F7attB50 is shown in detail. The hypersensitive sites “*”, HS1, and HS2 are shown as gray boxes. HS3, which comprises the iab-7 PRE, is shown in blue. F7attB50 landing platform (shown below) contains an attP site for the integration of the tested constructs; lox and frt sites were used for excision of the plasmid body and of rosy maker gene. The plasmid that was injected into Fab-7attp50 line, contains attB site for integration, HS3 iab-7 PRE for restoring functional integrity of the iab-7 domain, frt sites for excision of rosy gene, rosy gene, lox sites for excision of the plasmid body (shown below). Testing elements were inserted just in front of iab-7 PRE. After integration of the plasmid within Fab-7attp50, ry+ transformants were selected. Then, rosy and plasmid cassette were excised by FLP-recombinase, to remove an about 10.2 kb additional sequence between the tested element and iab-7 in ry+ line. (TIF) [file pgen.1006188.s004.tif]
