# Supplementary material for: Functional Dissection of the Blocking and Bypass Activities of the Fab-8 Boundary in the Drosophila Bithorax Complex
Source: PLoS Genet. 2016 Jul 18;12(7):e1006188. doi: 10.1371/journal.pgen.1006188 (PMC4948906; doi:10.1371/journal.pgen.1006188)
Supplement: S3 Fig — The molecular map of the F8337 insulator and F8CTCF Dir-Rev and F8CTCF Dir-Dir is the same as in S2 Fig. The PTS sequence is highlighted with gray. Inverted dCTCF binding sites are highlighted with yellow. Elba binding sites are in orange, GAF–in blue, dCTCF–in red. (PDF) [file pgen.1006188.s003.pdf]

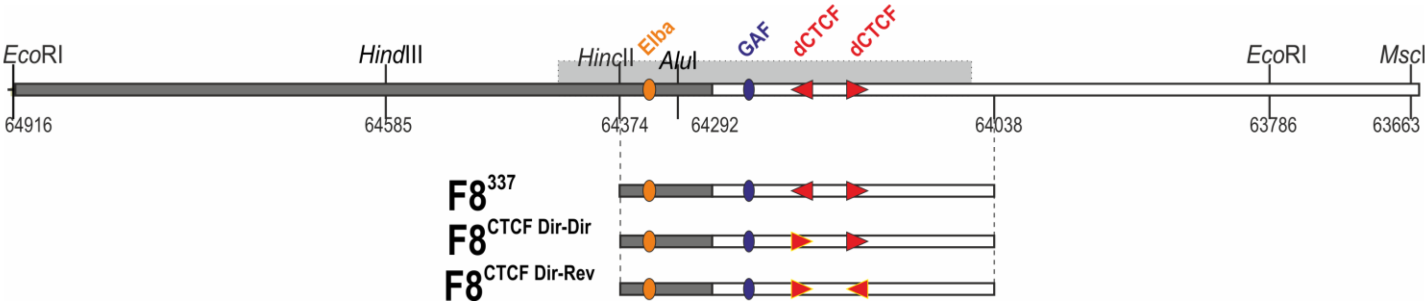

Fab8[337]

AACGCCAACCAGCACAAACACATTCGAATAAGACTCACTCACACAGTGGCAAGCTGTGCAAGGCATTTGTGTTGGTGAGCAAGCG
AAGAGTTCCATTCTCTGCTTCGAAGTACGAAGAGAGAAAGAGTACTTTAAATTTCCACATTCCCGCCTTGCAGCGCCACCTGGCCT
TGGTAATGTAGAACTAGGAAGGAAAGCACCAACACAAGATGTCGCTCTCCGACAGTGGACATGTCGCGTAAAAAATGTTCGATAAC
TTTCAATGGTTCGATTGAACAGACAATAAGTGTATTTAAGACACCAGTTCTTATATTCAAAAATCCTAACAACTCACATT

Fab-8[CTCF Dir-Dir]

AACGCCAACCAGCACAAACACATTCGAATAAGACTCACTCACACAGTGGCAAGCTGTGCAAGGCATTTGTGTTGGTGAGCAAGCG
AAGAGTTCCATTCTCTGCTTCGAAGTACGAAGAGAGAAAGAGTACTTTAAATTTCCACATTCCCGCCTTGCAGCTTACCAAGGCCA
GGTGGCGCTGCAATAATGTAGAACTAGGAAGGAAAGCACCAACACAAGATGTCGCTCTCCGACAGTGGACATGTCGCGTAAAAAA
TGTTCGATAACTTTCAATGGTTCGATTGAACAGACAATAAGTGTATTTAAGACACCAGTTCTTATATTCAAAAATCCTAACAACTCAC
ATT

Fab-8[CTCF Dir-Rev]

AACGCCAACCAGCACAAACACATTCGAATAAGACTCACTCACACAGTGGCAAGCTGTGCAAGGCATTTGTGTTGGTGAGCAAGCG
AAGAGTTCCATTCTCTGCTTCGAAGTACGAAGAGAGAAAGAGTACTTTAAATTTCCACATTCCCGCC**TT**GCAGCTTACCAAGGCCA
GGTGGCGCTGCAATAATGTAGAACTAGGAAGGAAAGCACCAAGTCGGAGAGCGACATCTTGTGTTCGACAGTGGACATGTCGCGT
AAAAAATGTTCGATAACTTTCAATGGTTCGATTGAACAGACAATAAGTGTATTTAAGACACCAGTTCTTATATTCAAAAATCCTAACAA
CTCACATT

Designations: PTS; Elba bs; GAF bs; CTCF bs; inverted sequence
